# A head-mounted photoacoustic fiberscope for hemodynamic imaging in mobile mice

**DOI:** 10.1038/s41377-024-01454-w

**Published:** 2024-05-07

**Authors:** Xiaoyan Zheng, Shuai Na

**Affiliations:** 1 National Biomedical Imaging Center, College of Future Technology, Peking University, Beijing, 100871 China; 2 Academy for Advanced Interdisciplinary Studies, Peking University, Beijing, 100871 China

**Keywords:** Interference microscopy, Photoacoustics

## Abstract

A miniaturized photoacoustic fiberscope has been developed, featuring a lateral resolution of 9 microns and a lightweight design at 4.5 grams. Engineered to capture hemodynamic processes at single-blood-vessel resolution at a rate of 0.2 Hz, this device represents an advancement in head-mounted tools for exploring intricate brain activities in mobile animals.

## Abstract (web summary)

A head-mounted photoacoustic fiberscope enables hemodynamic imaging in mobile mice.

## Graphical abstract

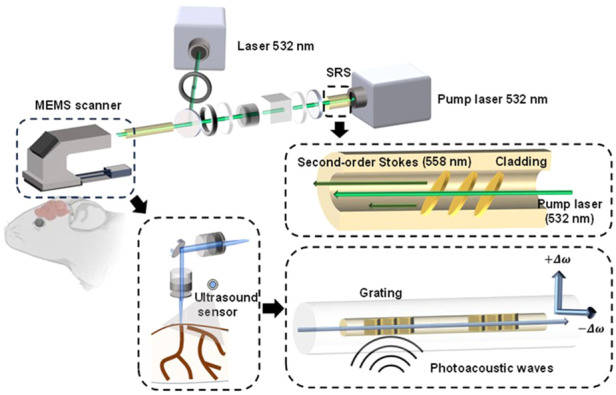

Blood transports oxygen and nutrients to the brain, supporting energy production and ensuring optimal brain function^1,2^. Monitoring cerebral oxygenation is essential in preventing and treating related disorders^3^. Photoacoustic imaging leverages hemoglobin’s (Hb’s) property of absorbing electromagnetic waves and generating acoustic waves via the photoacoustic effect, allowing for the quantification of oxyhemoglobin and deoxyhemoglobin concentrations based on their distinct absorption coefficients^4–7^. Yet, applying existing photoacoustic microscopes is largely restricted to sedated animals, while anesthesia can disrupt normal brain metabolism and vascular function^8^. Miniaturized single-photon and multi-photon microscopes have recently emerged as pivotal technologies for recording cortical neuron activities in mobile mice^9–12^. Nevertheless, these modalities inadequately measure brain hemodynamics and oxygenation levels. Although several miniaturized photoacoustic probes have been reported, they lack either a high spatiotemporal resolution or a superior sensitivity, underscoring the need for further development^13^.

Addressing these limitations, the research team led by Professors Guan Baiou and Long Jin at Jinan University has unveiled an optical fiber-based photoacoustic microscope^14^. The highlight of their invention, as illustrated in Fig. 1, is the compact imaging probe, weighing merely 4.5 grams, which encompasses two optical fibers for dual-wavelength photoacoustic excitation and detection. The excitation light, comprising a 532 nm wavelength and a 558 nm stimulated raman scattering wavelength, initiates photoacoustic waves. These waves induce deformation of the detecting laser cavity, causing the optical fiber propagation to exhibit birefringence. Subsequently, two laser modes in orthogonal polarization states undergo a frequency shift; the magnitude of change remains identical, albeit with opposite signs. Finally, an optical heterodyne interference technique is employed to capture the signal output. Photoacoustic sensing has relied predominantly on focused piezoelectric ultrasonic transducers, with inherent limitations in size, sensitivity, and field of view^15,16^. The proposed probe utilizes an optical sensor to convert acoustic displacement into a phase delay or intensity variation of the laser beam, improving the sensitivity by two orders of magnitude. Moreover, the widefield detection strategy eliminates the necessity for maintaining confocal between the excitation laser beam and the detection field, simplifying the scanning process^17^.

**Fig. 1:**
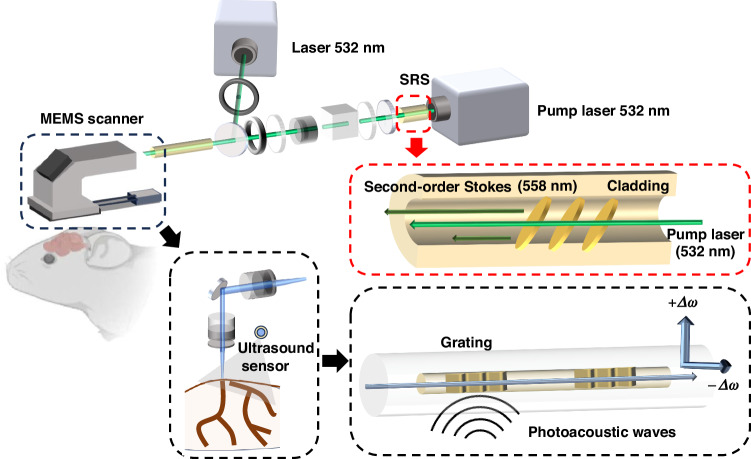
Schematic diagram of the head-mounted photoacoustic fiberscope

The imaging probe was affixed to the mouse head, allowing the visualization of blood vessels as the subject transitioned from a state of anesthesia to wakefulness, as displayed in Fig. 2a. Throughout this transition, the cerebral arteries exhibited constriction and an increase in oxygen saturation (sO_2_), indicating that the cerebral vasculature in awake mice increased oxygen transport to counteract the effects of hypercapnia—a response that was not observed in anesthetized mice. The probe was also used to explore the oxygenation dynamics in a hypercapnia cycle, revealing similar trends in oxygen saturation (Fig. 2b) and hemoglobin (Fig. 2c) levels across both arteries and veins. Additionally, in exploring the effect of hyperlipidemia on cerebral metabolism, measurements of the blood vessel diameter, among other parameters, indicated that obese mice exhibited a diminished ability to adjust oxygen supply in response to external stimuli. These experimental results aligned with preceding medical literature and theories established by benchtop photoacoustic microscopy^14^.

**Fig. 2:**
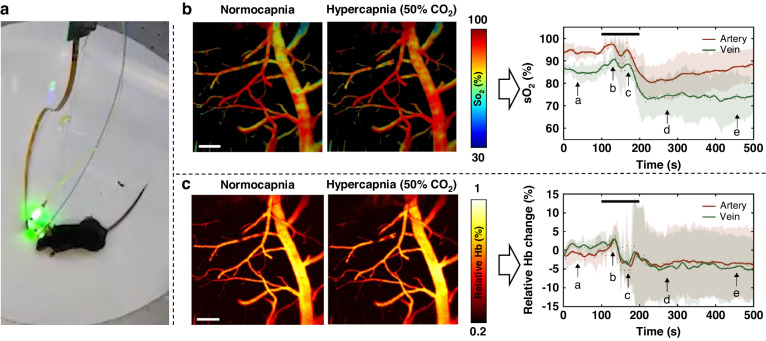
Experimental results of the hypercapnia challenge. **a** A freely moving mouse wearing the imaging probe; **b** sO_2_ changes in a hypercapnia-normocapnia cycle; **c** The corresponding changes in Hb levels

The developed photoacoustic fiberscope showcases remarkable imaging capabilities; however, there is room for further enhancement. The microscope achieves a lateral resolution of 9 µm and a longitudinal resolution of 165 µm, limitations of which are due in part to the acoustic impedance mismatch between the optical fiber and the ambient coupling medium. The imaging depth is additionally limited by the relatively short wavelengths, which reduces the penetration due to the more severe tissue scattering and absorption. Adopting near-infrared wavelengths could enhance the imaging depth, achieving penetration on the scale of millimeters^18^. Overall, this device holds immense potential for advancing disease monitoring and recovery processes following major surgeries. Future upgrades are expected to possibly integrate with other imaging techniques, laying the groundwork for wide-ranging brain research.
